# LSD1 mediated changes in the local redox environment during the DNA damage response

**DOI:** 10.1371/journal.pone.0201907

**Published:** 2018-08-10

**Authors:** Michelle L. Duquette, Justine Kim, Linda Z. Shi, Michael W. Berns

**Affiliations:** 1 Institute of Engineering in Medicine, University of California, San Diego, CA, United States of America; 2 Department of Bioengineering, University of California, San Diego, CA, United States of America; 3 Beckman Laser Institute, University of California, Irvine, Irvine, CA, United States of America; University of South Alabama Mitchell Cancer Institute, UNITED STATES

## Abstract

The redox state of the cell can be affected by many cellular conditions. In this study we show that detectable reactive oxygen species (ROS) are also generated in response to DNA damage by the chromatin remodeling factor and monoamine oxidase LSD1/KDM1A. This raised the possibility that the localized generation of hydrogen peroxide produced by LSD1 may affect the function of proximally located DNA repair proteins. The two major pathways for repair of DNA double-strand breaks (DSBs) are homologous recombination (HR) and non-homologous end joining (NHEJ). Cells were exposed to low levels of ectopic H_2_O_2_, DNA breaks generated by laser light, and recruitment kinetics of NHEJ protein Ku80 to DNA damage sites determined. Ku80 recruitment to damage sites was significantly decreased in cells pretreated with H_2_O_2_ while HR end binding protein Nbs1 was increased. This suggests that the DNA repair pathway choice has the potential to be modulated by the local redox state. This has implications for chemotherapeutic approaches involving generating DNA damage to target actively dividing cancer cells, which may be more or less effective dependent on the redox state of the targeted cells and the predominant repair pathway required to repair the type of DNA damage generated.

## Introduction

The redox state of the cell can be affected by many conditions including the level of cellular respiration [1], cellular activation [2], environmental exposure to oxidizing agents [3] and disease [4]. A change in the redox environment within a cell can trigger redox sensitive signaling cascades [5]. Redox sensitive thiols present on proteins can act as regulatory switches [5–8] and activate cell signaling cascades leading to modifications in cellular function at multiple levels from regulating the actin cytoskeleton [9] to the level of transcription [10].

The DNA damage response involves the activation of multiple pathways dependent on the type of the initial DNA lesion. However, not much is known about redox sensitive DNA damage signaling. One type of DNA damage involving base damage commonly caused by base oxidation and the repair pathway, base excision repair (BER), involves Ape1 a known redox regulated factor [11]. In addition, it has been shown the Ku80 a Non-homologous end joining factor that binds DNA ends has been found to have oxidation sensitive DNA binding *in vitro* [12, 13]. There are also histone modifiers that are oxidases and whose enzymatic reaction chemically produces the release of hydrogen peroxide as a byproduct. LSD1 is a flavin adenine dinucleotide (FAD)-dependent amine oxidase that demethylates histone H3 Lys4 (H3-K4) [14]. The demethylation of histone H3 Lys4 provides a transcription regulatory function as well as being shown to be recruited to sites of DNA damage involving double strand breaks [15]

We asked whether there was a redox based involvement in the DNA damage response to double strand breaks. Region specific DNA breaks can be created in single cells using laser light that generates DNA double strand breaks [16] that damages DNA but does not directly generate reactive oxygen species (ROS). Using a combination of ROS specific dyes and monoamine oxidase inhibitor we have found that the oxidase and chromatin remodeling protein Lysine demethylase I (LSD1/KDM1A) generates detectable ROS as a byproduct of its chromatin remodeling activity during the initial DNA damage response. ROS is produced at detectable amounts primarily within the first 3 minutes post irradiation. There are many cellular functions that are known to be regulated by the redox state of the cell and controlled by the oxidation or reduction of cysteine residues. Here we show how proteins that function in parallel double strand break repair pathways are affected by oxidizing conditions. These data reveal a novel source of reactive oxygen species that is associated with the DNA damage response and has implications for double strand DNA repair pathway choice. Many chemotherapeutic approaches kill actively dividing cancer cells by generating DNA damage. These data have implications for chemotherapeutic approaches involving generating DNA damage to target actively dividing cancer cells, which may be more or less effective dependent on the redox state of the targeted cells and the predominant repair pathway required to repair the type of DNA damage generated.

## Results and discussion

We began by utilizing an inducible system to generate region specific double strand breaks (DSBs) in live cells to study the DNA damage response [17]. PPOI is an endonuclease that recognizes and cleaves a specific DNA sequence present in the rDNA. PPOI is fused to an estrogen receptor that translocates into the nucleus upon addition of tamoxifen (4-OHT). Upon induction of DSB generation following addition of 4-OHT the DNA damage response can be monitored (S1A Fig). Upon induction of PPOI, markers of the DNA damage response accumulated at the rDNA containing nucleoli in human osteosarcoma (U2OS) cells. DNA damage response proteins γH2AX and BRCA1 only accumulated at the nucleoli in the presence of both PPO1 and 4-OHT when DNA damage was induced. To our surprise, laser generated double strand breaks (DSBs) also resulted in the accumulation of the base excision repair protein Ogg1 which recognizes and binds to the oxidized nucleic acid 8-oxo-guanine [18] (S1B Fig). The accumulation of Ogg1 suggested that the reactive oxygen species could potentially be generated during the DNA damage response.

**Fig 1:**
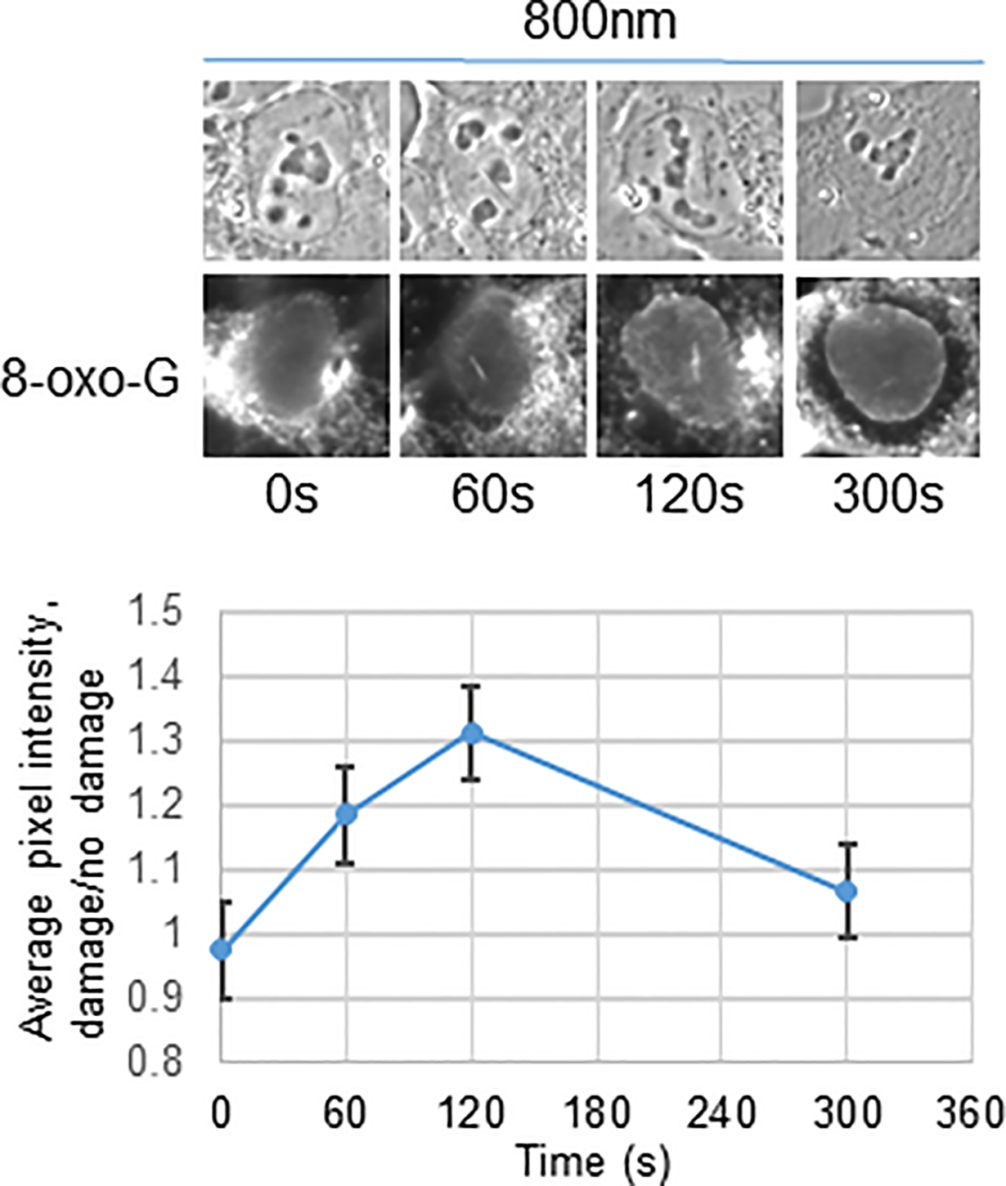
8-oxo-guanine accumulates at damage sites following laser irradiation due to ROS generation. A. DNA damage was created by 730 nm light (left) and 800nm light (right) from a femtosecond near infrared (NIR) laser. Cells were then fixed at different time points post irradiation and stained at with antibody against 8-oxo-guanine and visualized. Quantification of 8-oxo-G was determined by dividing pixel intensity of fluorescence along laser cut by background (uncut region). B. U2OS cells were treated with ROS sensing dye and irradiated with 60mW 800 nm light (above) 50 mW 730 nm light (below) from a femtosecond near infrared (NIR) laser. Live cells were followed over time pre and post irradiation and the ratio of the pixel intensity of the reacted dye over background was calculated. Pixel intensity of dye fluorescence (higher intensity indicates presence of ROS) along laser track divided by background was calculated to determine the kinetics of dye reaction following laser irradiation.

We next examined whether oxidative damage was generated at the sites of DNA double strand breaks using an alternate approach. We determined conditions in which region specific DNA breaks could be created in single cells using pulsed (fs) near infra-red (800 nm) laser light. Different laser systems are commonly employed for the study of the DNA damage response. We chose NIR 800nm because it generates the highest density of DSBs and unlike UVA lasers will produce less ROS and thus additional base damage [19]. A UVA laser or other chemicals commonly used to generate dsbreaks would directly generate ROS thus potentially mask any secondary release of ROS by the cellular machineryIf free radicals were being released at irradiated sites post laser irradiation, one would expect the DNA in the vicinity to be oxidized and damaged. Therefore we examined the kinetics of oxidative DNA damage generation by visualizing 8-oxo-guanine accumulation along irradiated tracks in U2OS cells. Cells were maintained at 37°C, 5% CO_2_, irradiated then fixed and stained for 8-oxo-guanine at different time points post irradiation at 60mW (1.27 X10^12^ W/cm^2^) Following irradiation at 800nm there is little to no immediate 8-oxo-G visible, but it accumulates over time, peaks at approximately 2 minutes, and beings to decrease (n = 15, representative cells show in Fig 1A). This suggests that the resulting oxidative DNA damage results indirectly from the laser and likely is created by reactive oxygen species generated indirectly by the cell as part of the DNA damage response.

To examine directly whether reactive oxygen species were being generated at sites of DNA damage we assayed for ROS production by pre-treating cells with a ROS sensitive dye (Enzo Life sciences) then irradiated with either 50mW (1.06 X10^12^ W/cm^2^) 730nm or 60mW (1.27 X10^12^ W/cm^2^) 800nm laser light. 730nm was used as a positive control for ROS production since 2-photon absorption at that wavelength is known to cause UV damage via the production of free radicals [20]. Cells were maintained at 37°C and 5% CO2 and images were acquired of live cells. Pixel intensity of dye fluorescence divided by background was calculated to determine the kinetics of dye reaction following laser irradiation. Immediately following irradiation there is a jump in detectable ROS in 730nm irradiated cells, and minimal amounts in 800nm cells (Fig 1B, B n = 20 for both). However, it was observed that there was a gradual increase in ROS as detected by the dye following irradiation at both wavelengths which suggested ROS continued to be produced following laser-induced DNA damage.

**Fig 2:**
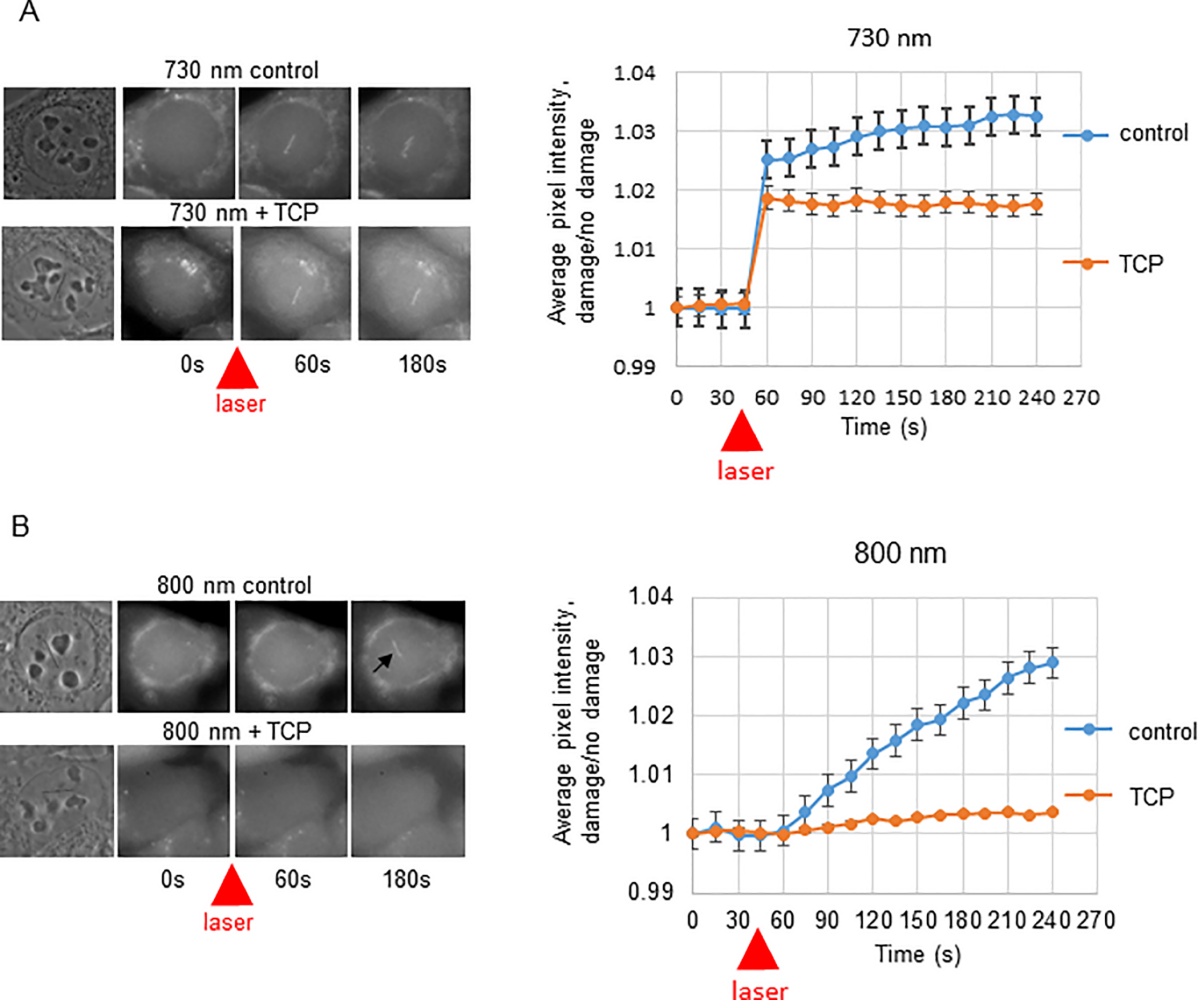
Mono-amine oxidase inhibition blocks delayed generation of ROS at sites of laser generated DNA damage. A. U2OS cells were treated with ROS sensing dye and irradiated with 50mW 730 nm light (above) or 60 mW 800 nm light (below) from a femtosecond near infrared (NIR) laser and either mock treated or treated with 2 μM of the monoamine oxidase tranylcypromine. Live cells were followed over time pre and post irradiation and the ratio of the pixel intensity of the reacted dye over background was calculated. Pixel intensity of dye fluorescence (higher intensity indicates presence of ROS) along laser track divided by background was calculated to determine the kinetics of dye reaction following laser irradiation.

In order to identify which factor(s) could be potentially responsible for the damage induced ROS generation we looked for potential nuclear oxidases. We began by looking at nuclear mono-amine oxidases which produce H_2_O_2_, a potential source for ROS. To address whether a mono-amine oxidase was responsible for the ROS accumulation at sites of laser generated DNA damage, the monoamine oxidase inhibitor tranylcypromine (TCP), a potent LSD1 inhibitor [21], was added to U2OS cells at 2μM or cells were mock treated with DMF (the TCP diluent). ROS reactive dye was then added, and cells irradiated with either 730 nm and 800nm laser light and assayed for ROS formation (Fig 2A and 2B n = 25 cells for each condition tested). The immediate increase in reacted ROS sensing dye following 730nm irradiation was unaffected by pretreatment with the monoamine oxidase inhibitor tranylcypromine while the slow increase in ROS post irradiation at both wavelengths was completely abolished by the oxidase inhibitor. This indicated that the gradual accumulation of ROS detected in 800nm in laser irradiated cells is generated indirectly by a cellular oxidase, while the immediate accumulation of ROS at 730nm is primarily a result from the laser irradiation itself.

**Fig 3:**
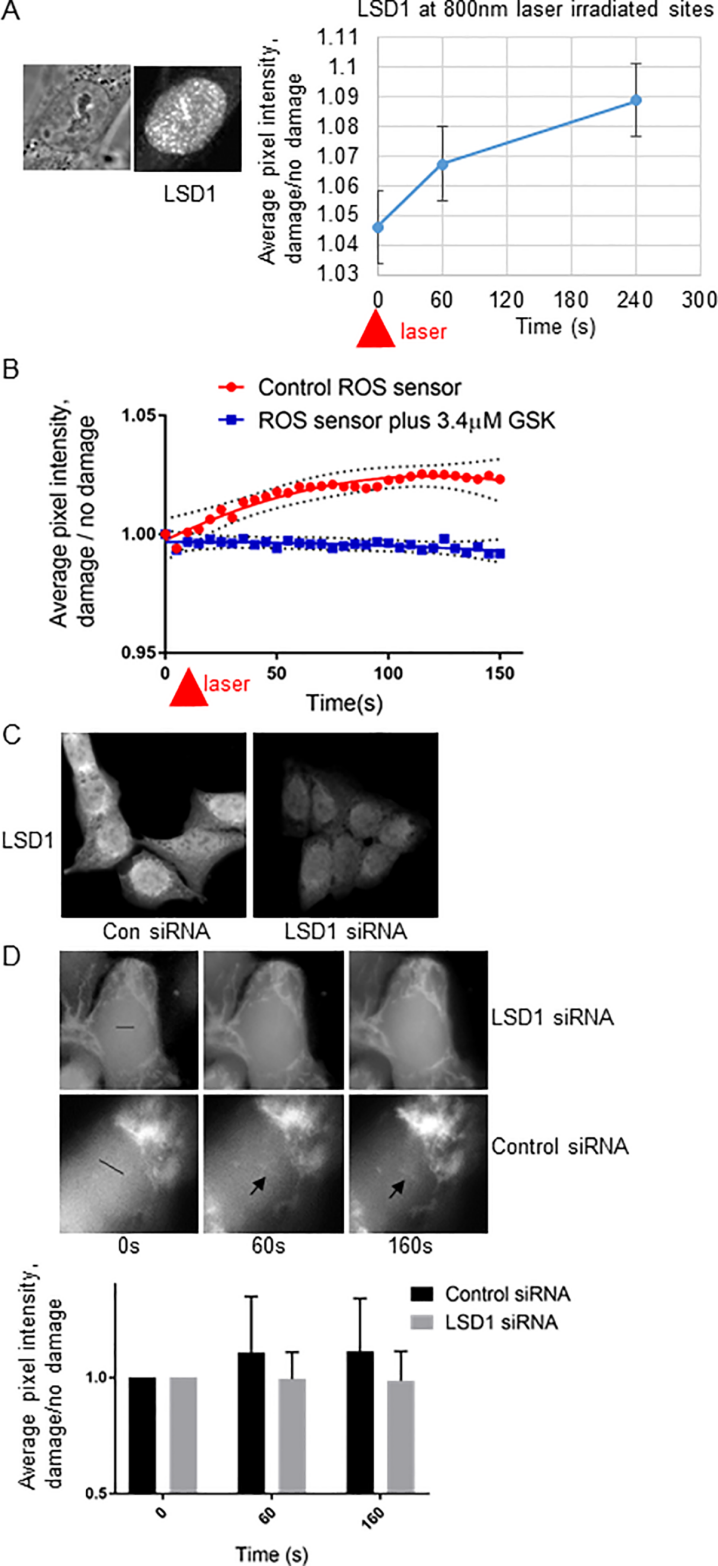
LSD1 accumulates at damaged sites and releases ROS upon formation of DNA damage. A. U2OS cells were irradiated with 800 nm laser light, fixed at different time points post irradiation then immunostained for LSD1. Quantification of LSD1 was determined by dividing pixel intensity of fluorescence along laser by background (uncut region). B. U2OS cells were treated with ROS sensing dye and irradiated with 800 nm light from a femtosecond near infrared (NIR) laser and either mock treated or treated with 3.4 μM of the LSD1 inhibitor GSK2879552. The pixel intensity along the laser track was quantified and divided by the background nuclear signal. The 95% confidence interval are shown above and below the best fit line for each data set. C. U2OS stained with antibody against LSD1 in either control siRNA treated cells or siLSD1 treated cells 36 hours post transfection. LSD1 expression as determined by IF was reduced approximately 39% in siLSD1 treated cells. D. Accumulation of ROS as sensed by ROS sensing dye in sicontrol treated cells vs siLSD1 treated cells. Pixel intensity of dye fluorescence (higher intensity indicates presence of ROS) along laser track divided by background was calculated to determine the kinetics of dye reaction following laser irradiation.

LSD1 is a flavin dependent mono-amine oxidase and histone demethylase which can demethylate mono- and di-methylated lysines on histone 3 (H3K4 and H3K9) [14]. As an oxidase it produces hydrogen peroxide as a byproduct of the flavin dependent demethylase activity. Its demethylation activity has been shown to be important for transcriptional regulation and recently for the DNA damage response [15, 22, 23]. While the expression of LSD1 peaks in S and G2 it actively demethylates chromatin in all stages of the cell cycle and has been found to oxidize DNA which can in turn affect transcriptional regulation [24–26]. LSD1 is one of the few oxidases and candidate for ROS production in the nucleus. Since LSD1 is an oxidase that has been implicated in the DNA damage response we examined the kinetics of its accumulation at laser irradiated sites. U2OS cells were laser irradiated with 800 nm laser light, fixed at different points post irradiation and stained with antibodies against LSD1. The kinetics of LSD1 accumulation at 800nm irradiated cells was similar to that of detectable ROS and 8-oxo-G suggesting that the detectable ROS observed at DNA damage sites was LSD1 dependent (Fig 3A). To follow up this observation we tested whether the generation of detectable ROS along laser irradiated sites could be blocked by adding a specific LSD1 inhibitor. We used GSK2979552 (GSK) which specifically and irreversibly inhibits LSD1 and is currently in clinical trials for [27] blocking LSD1 activity in tumors [28]. Cells were treated with 3.4 μM GSK or mock treated prior to irradiation, the ROS fluorescent oxidative sensor added, then cells were irradiated and fluorescence monitored. The gradual increase in ROS post irradiation at 800nm was completely abolished by the LSD1 inhibitor GSK (Fig 3B). We also examined whether depletion of LSD1 by siRNA was able to reduce ROS generation at 800nm laser irradiated tracks. U2OS cells were transfected with either LSD1 specific siRNA or non-specific control siRNA and cells assessed for ROS generation along irradiated tracks as previously described 36 hours post-transfection. LSD1 levels were confirmed to be reduced in siLSD1 transfected cells by 39% as assayed by LSD1 immunostaining (Fig 3C). High levels of LSD1 depletion affects G1 transition therefore we chose conditions that only partially reduced LSD1 expression [27]. ROS generation along laser irradiated tracks was found to be reduced in siLSD1 treated cells as compared to sicontrol cells (Fig 3D n = 25 cells for each condition tested). The combination of inhibitor and LSD1 depletion data indicated that the gradual accumulation of ROS detected in 800nm in laser irradiated cells is generated indirectly by LSD1.

**Fig 4:**
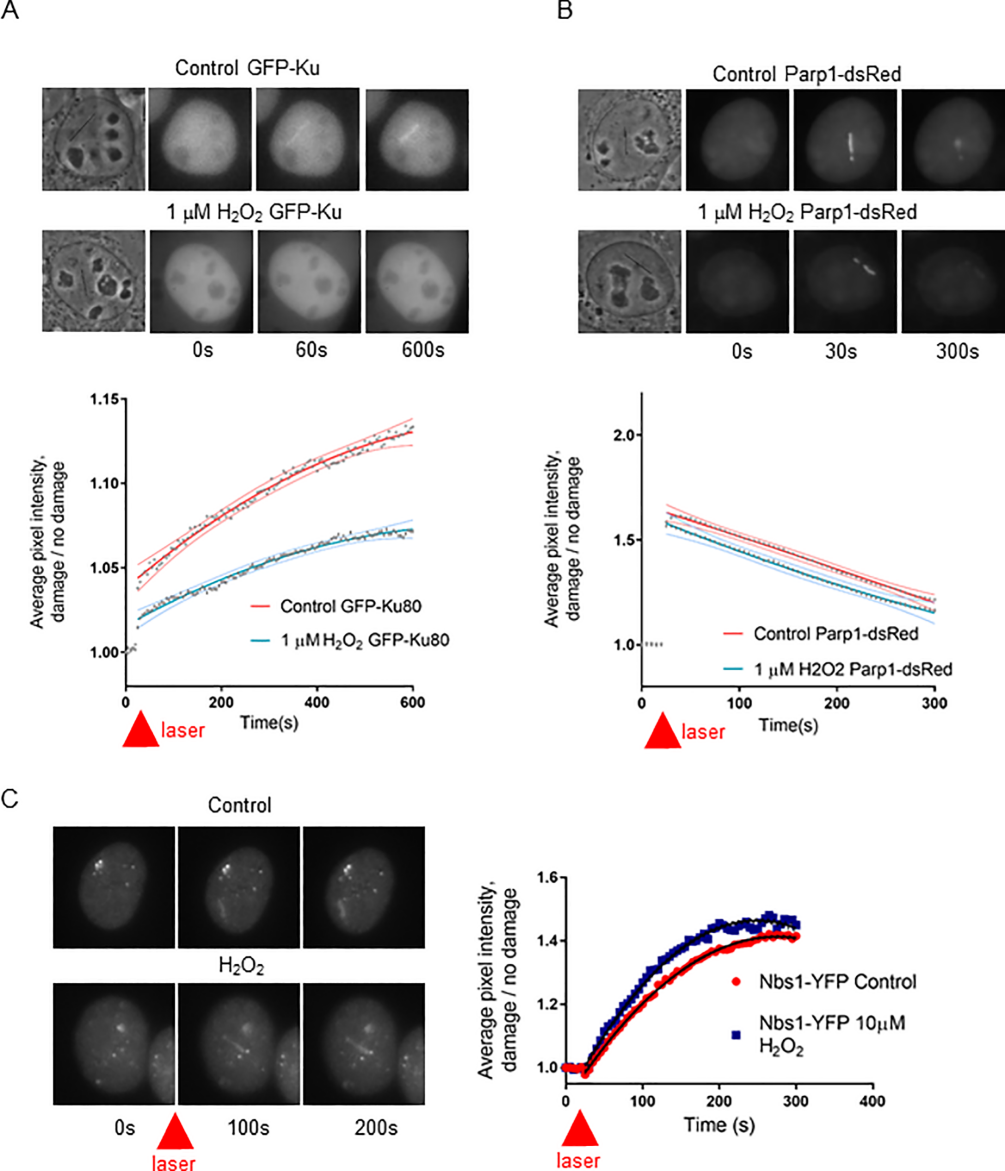
An oxidizing environment increases Nbs1 recruitment and reduces Ku80 recruitment to sites of DNA damage. **A**. U2OS cells were either untreated (control) or treated with 1μM H_2_O_2_ then irradiated and GFP-Ku recruitment tracked. The pixel intensity along the laser track was quantified and divided by the background nuclear signal. The 95% confidence interval are shown above and below the best fit line for each data set. **B**. U2OS Cells were either untreated (control) or treated with 1μM H_2_O_2_ then irradiated and Parp1-dsred recruitment tracked. The pixel intensity along the laser track was quantified and divided by the background nuclear signal. The 95% confidence interval are shown above and below the best fit line for each data set. **C**. U2OS Cells were either untreated (control) or treated with 10μM H_2_O_2_ then irradiated and Nbs1-YFP recruitment tracked. The pixel intensity along the laser track was quantified and divided by the background nuclear signal. The 95% confidence interval are shown above and below the best fit line for each data set.

The two major pathways for repair of DNA double-strand breaks (DSBs) are homologous recombination (HR) and nonhomologous end joining (NHEJ). Ku70/80 heterodimer is a DNA end binding protein complex involved in NHEJ. Previously published studies have demonstrated that the DNA end binding protein Ku80 is redox sensitive. Its binding to DNA ends *in vitro* is reduced in an oxidizing environment and predicted to be dependent on cysteines positioned near the DNA binding domain [12, 13]. To examine how Ku80 accumulates at sites of DNA damage under very low oxidizing conditions we exposed cells to low levels of ectopic H_2_O_2_, 1μM for 10 minutes, then generated DNA breaks by laser light, and assayed the kinetics of Ku80-GFP recruitment to the damage sites. Ku80 recruitment to damage sites was significantly decreased in cells pretreated with H_2_O_2_ (Fig 4A n = 20 per condition). Next we examined recruitment of Parp1-dsred under similar conditions (Fig 4B n = 20 per condition). Parp1 and Ku80 have been shown to compete for DNA ends and affect the balance between high fidelity and low fidelity repair [29] [16]. In contrast to Ku80, Parp1 DNA end binding was not affected by 1μM H_2_O_2_. We next examined Nbs1-YFP recruitment under normal and oxidizing conditions. Also in contrast to Ku80, HR end binding protein Nbs1 [17] had a slight increase in recruitment in the presence of 10μM H_2_O_2_ (Fig 4C n = 15 per condition). This suggests that the DNA repair pathway choice has the potential to be modulated by the local redox state. Under oxidizing conditions, HR is the preferred pathway which LSD1 can potentially facilitate via its oxidase function.

## Conclusion

While reactive oxygen species are usually associated with damaging DNA, we have found that it is created at detectable levels as part of the DNA damage response. Using a combination of ROS specific dyes, monoamine oxidase inhibitor, LSD1 inhibitor, and LSD1 depletion by siRNA we have found that the oxidase and chromatin remodeling protein Lysine demethylase I (LSD1/KDM1A) generates detectable ROS as a byproduct of its chromatin remodeling activity during the initial DNA damage response. This is the first evidence showing that significant levels of ROS are produced as a byproduct of the DNA damage response. There are many cellular functions that are known to be regulated by the redox state of the cell and controlled by the oxidation or reduction of cysteine residues. These data reveal a novel source of reactive oxygen species associated with the DNA damage response.

**Fig 5:**
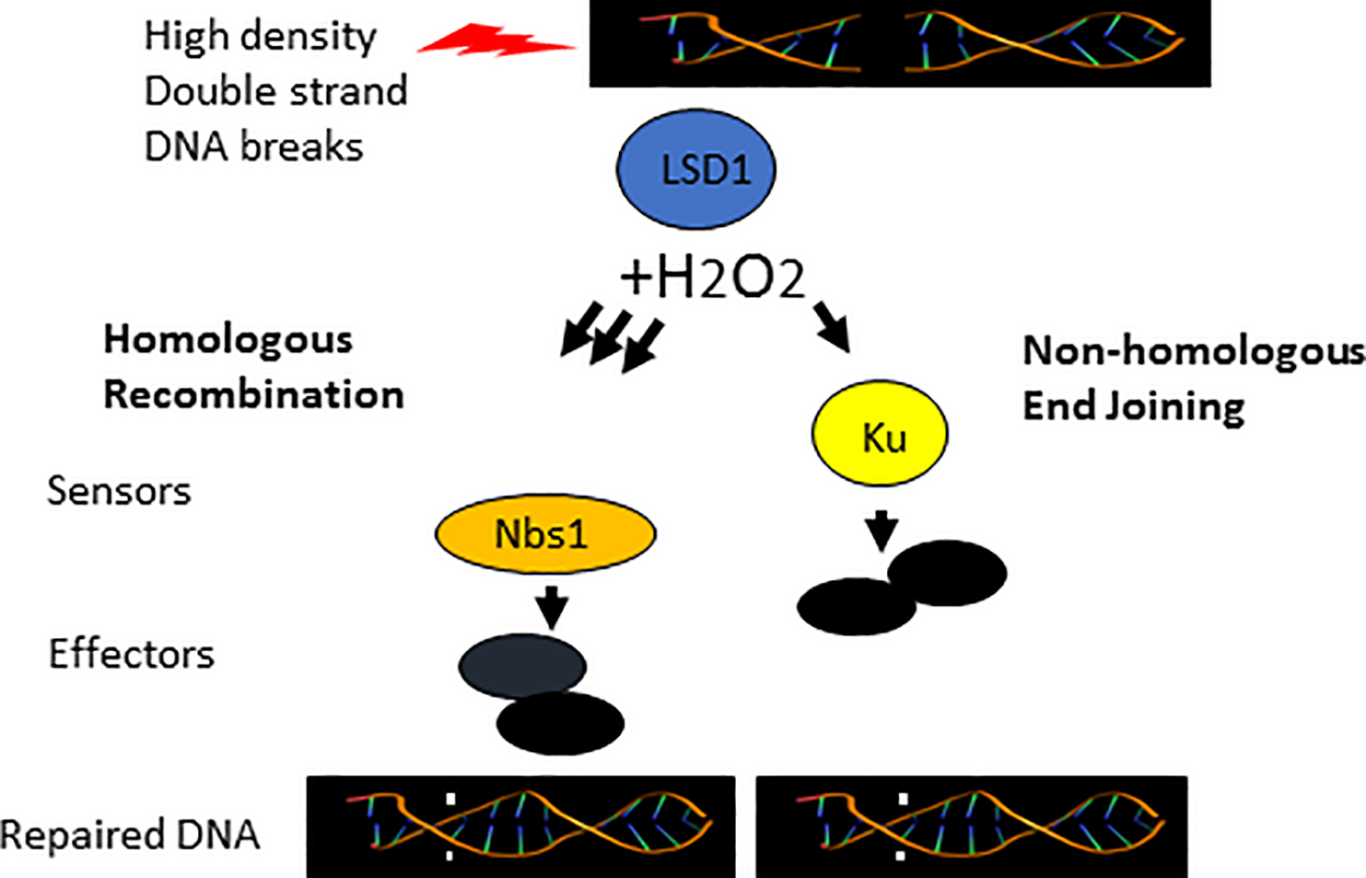
High density generation of dsBreaks leads to an LSD1 dependent increase in local oxidative environment favoring DNA damage response initiation by Nbs1. LSD1 is recruited to double strand breaks. Its histone demethylase activity results in H_2_O_2_ as a byproduct. H_2_O_2_ reduces DNA end binding activity of Ku80 favoring Nbs1 and HR.

Our data suggests that an oxidizing environment favors dsbreak end binding by the sensor and HR factor Nbs1 over NHEJ factor Ku80. This is likely due to the high density of double strand breaks generated by high power NIR radiation resulting in a high density of LSD1 binding and chromatin demethylation activity. This in turn results in a local release of H_2_O_2_ from LSD1 at a level that destabilizes Ku80’s redox sensitive end binding activity thus favoring binding by Nbs1 [12, 13]. (Fig 5). It remains to be determined if there is a critical level of LSD1 dependent H_2_O_2_, potentially modulated by the density of DSBs and/or mixture of DNA lesions that can affect the recruitment of DNA damage sensor proteins and repair pathway choice. Although the recruitment of Ku80 and Nbs1 were affected by the local redox state, we observed that Parp1 binding was minimally affected. Although Parp1 binding to damage sites was minimally affected by the oxidative state, this does not preclude other oxidation sensitive Parp1 interacting factors such as PolQ from affecting the DNA damage response [30].

Further studies will be aimed at elucidating the effects of whole cell changes in redox state under medically relevant conditions, such as during an inflammatory response, on DNA repair pathway choice. The data presented here has implications for chemotherapeutic approaches involving the generation of DNA damage to target actively dividing cancer cells, which may be more or less effective dependent on the redox state of the targeted cells and the predominant repair pathway required to repair the type of DNA damage generated. Ideally a chemotherapeutic approach utilizing DNA damage to target actively dividing cancer cells would generate a type of damage least likely to be repaired given the predominant functioning repair pathway in that cell type which may be modulated by the existing oxidative environment.

## Materials and methods

### PPOI induced double strand nucleolar breaks

U2OS cells were treated as described by [17].

#### Laser irradiation and imaging

Cells for laser microirradiation were cultured on non-gridded (live cell imaging) or gridded glass bottom dishes (for indirect immunostaining) (Mattek, Ashland, MA). Laser irradiation and imaging was performed on a tunable (690–1040 nm) femtosecond mode locked Ti:Sapphire infrared laser (Mai Tai, Spectraphysics, Newport Corp., Mountain view, CA) coupled to microscope allowing targeting of subcellular regions [31]. The laser was used at 730nm or 800nm (effective wavelength 365nm or 400nm via 2-photon excitation [20] Laser power was 50mW (for 730nm) and 60mW (for 800nm) before entering the 63X 1.4 NA phase contrast objective. The peak irradiance at the focal point was 1.06 X10^12^ W/cm2 for 730nm and 1.27 X10^12^ W/cm^2^ for 800nm [31]. Cells treated with LSD1 inhibitor GSK2879552 (Chemietek, Indianapolis, IN) were incubated in HBSS containing 3.4μM GSK inhibitor for 1 hour at 37°C prior to irradiation. Experiments involving GSK were carried out with a peak irradiance of 1.27 X10^12^ W/cm2 at 800nm with a series of 6.97 X10^-7^ m sized irradiated spots. Cells treated with H_2_O_2_ were washed with HBSS (Thermo Fisher Scientific, Waltham, MA) and HBSS containing either 1μM or 10μM H_2_O_2_ was added 10 minutes prior to irradiation. Cells incubated with tranylcypromine (Tocris, Bristol, UK) were incubated with 2μM tranylcypromine at 37°C for 30 minutes prior to irradiation. U2OS Cells were maintained at 37°C and 5% CO2 during irradiation and imaging with an Ibidi stage heater. (Ibidi, Munich, Germany). To image reactive oxygen species, 1uM final concentration of the Oxidative Stress Detection Reagent from Enzo Life Sciences (Farmingdale, NY) was added to dishes in HBSS plus or minus HSK or TCP inhibitor for a half hour prior to irradiation.

#### Cell culture and transfection

Asynchronous or rapidly proliferating U2OS cells (ATCC) were maintained in Dulbecco's modified Eagle's medium (Invitrogen, Carlsbad, CA) supplemented with 10% bovine calf serum L-glutamine, and sodium pyruvate) at 37°C and 5% CO2. Cells were transfected with either pKu80-GFP, pParp1-dsred, or pNbs1-YFP CtIP using Effectene (Qiagen, Hilden, Germany). Experiments were performed 24 hours post transfection.

#### siRNA transfection

Small interfering double stranded RNAs (siRNAs) were introduced into U2OS cells by transfecting cells in a 6 well dish with 75 pmol siRNA, and 5 μL Dharmafect 1 (Dharmacon, Lafayette, CO) per reaction. The following synthetic siRNAs used were obtained from Thermofisher. Scientific: *Silencer*™ Negative Control No. 1 siRNA Cat. # AM4611, and **Silencer™ Pre-Designed siRNA** against LSD1 Cat. # AM4611. 24 hours post transfection cells were seeded onto glass bottom dishes (Mattek). Cells were assayed 36 hours post transfection.

#### Immunostaining

Cells were fixed in 3.7% para-formaldehyde phosphate buffered saline (PBS) at room temperature for 10 minutes, and permeabilized with 0.2% Triton-X. Cells were then stained with primary antibody in 3% BSA/PBS. LSD1 primary antibody was used at 1:200 #2139 (Cell Signaling Technology, Danvers, MA). Polyclonal rabbit LSD1 primary antibody used for confirming siRNA depletion was used at 1:100, Cat# PA511306 (Thermofisher Scientific). 8-oxo-dG staining. Fix in 4% paraformaldehyde at RT for 15 minutes, wash 2 X 5 minutes in PBS, 0.1% TritonX-100 10 min at RT, 100μg/mL RNase A for 1 hr at 37°C, 10μg /μL Proteinase K for 10 min at RT, 2M HCL for 5 min, Neutralize in 1M Tris pH 7.4 for 5 min, Block in 2% BSA/PBS for 1 hr at RT, Dilute anti-8-oxo-dG monoclonal antibody (Clone 2E2) Trevigen, Cat#4354-MC-1:100 in 3% BSA/PBS. For both LSD1 and 8-oxo-G Incubate at RT for 2 hours, Wash 2 X 5 min in 0.05% Triton-X-100, 2° anti-mouse 1:5000 (for 8-oxo-dG) 2° anti-rabbit 1:5000 (for LSD1) in 2% BSA/PBS for 2 hrs at RT, wash 2 x 5 min 0.05% Triton-X-100. Glass coverslips were mounted with Vectashield (Vectorlabs, Burlingham, CA).Samples were visualized and images acquired using a 63× objective on a Leica DM IRE2 microscope equipped with a Hamamatsu C4742-95 digital charge-coupled-device camera. LSD1 depletion efficiency was determined by measuring the mean pixel intensity in each cell (n = 50) in both siRNA and consiRNA containings cells.

## Supporting information

S1 Fig Ogg1 accumulates at region specific double strand breaks. S1A. B DNA damage response proteins γH2AX and BRCA1 only accumulate at the nucleoli in the presence of both endonuclease PPO1 and 4-OHT when DNA damage is induced in U2OS cells. S2B. Ogg1 accumulates in nucleoli upon expression of nucleolar PPOI.
(TIF)
