# Supplementary figures and images for: LSD1 mediated changes in the local redox environment during the DNA damage response
Source: PLoS One. 2018 Aug 10;13(8):e0201907. doi: 10.1371/journal.pone.0201907 (PMC6086436; doi:10.1371/journal.pone.0201907)

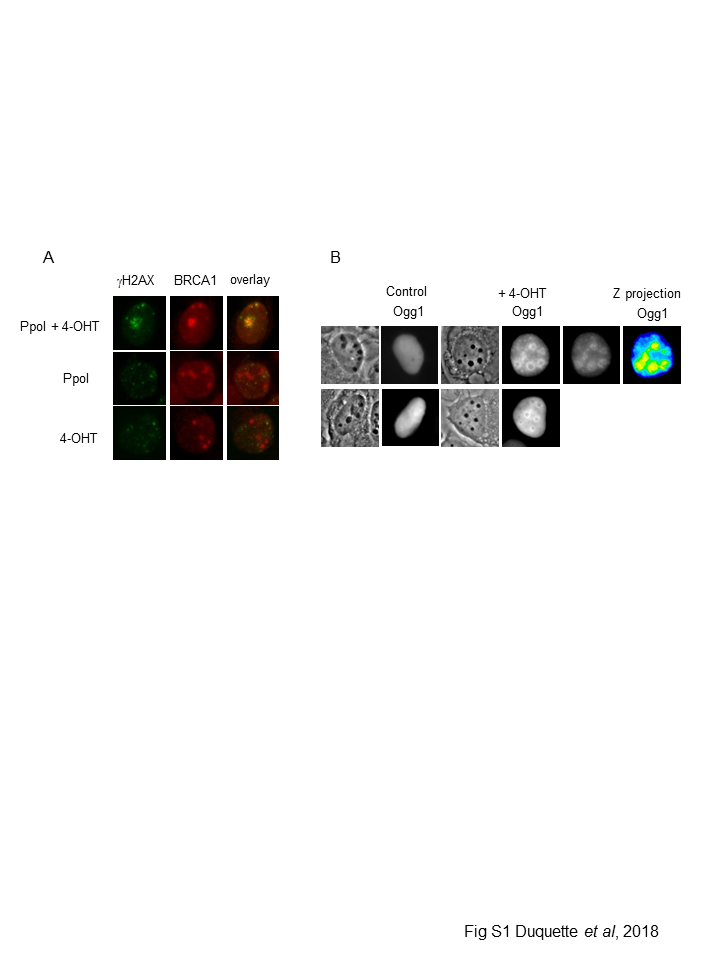

Supplement: S1 Fig — S1A. B DNA damage response proteins γH2AX and BRCA1 only accumulate at the nucleoli in the presence of both endonuclease PPO1 and 4-OHT when DNA damage is induced in U2OS cells. S2B. Ogg1 accumulates in nucleoli upon expression of nucleolar PPOI. (TIF) [file pone.0201907.s001.TIF]
